# Exploring the consequences of decentralization: has privatization of health services been the perceived effect of decentralization in Khartoum locality, Sudan?

**DOI:** 10.1186/s12913-020-05511-z

**Published:** 2020-07-20

**Authors:** Bandar Noory, Sara A. Hassanain, Benedikte Victoria Lindskog, Asma Elsony, Gunnar Aksel Bjune

**Affiliations:** 1 The Epidemiological Laboratory, Khartoum, Sudan; 2 International Community Health, University of Oslo, Oslo, Norway; 3 Federal Ministry of Health, Khartoum, Sudan; 4 Department of International studies and Interpreting, Oslo Metropolitan University, Oslo, Norway; 5 Department of Community Medicine, Institute of Health and Society, University of Oslo, Oslo, Norway

**Keywords:** Decentralization, Privatization, Stakeholders, Global liberalisation, Profit-making

## Abstract

**Background:**

The health system of Sudan has experienced several forms of decentralization, as well as, a radical reform. Authority and governance of secondary and tertiary health facilities have been shifted from federal to state levels. Moreover, the provision of health care services have been moved from large federal tertiary level hospitals such as Khartoum Teaching Hospital (KTH) and Jafaar Ibnoaf Hospital (JIH), located in the center of Khartoum, to smaller district secondary hospitals like Ibrahim Malik (IBMH), which is located in the southern part of Khartoum. Exploring stakeholders’ perceptions on this decentralisation implementation and its relevant consequences is vital in building an empirical benchmark for the improvement of health systems.

**Methods:**

This study utilised a qualitative design which is comprised of in-depth interviews and qualitative content analysis with an inductive approach. The study was conducted between July and December 2015, and aimed at understanding the personal experiences and perceptions of stakeholders towards decentralisation enforcement and the implications on public health services, with a particular focus on the Khartoum locality. It involved community members residing in the Khartoum Locality, specifically in catchments area where hospital decentralisation was implemented, as well as, affiliated health workers and policymakers.

**Results:**

The major finding suggested that privatisation of health services occurred after decentralisation. The study participants also highlighted that scrutiny and reduction of budgets allocated to health services led to an instantaneous enforcement of cost recovery user fee.

Devolving KTH Khartoum Teaching and Jafar Ibnoaf Hospitals into peripherals with less. Capacity, was considered to be a plan to weaken public health services and outsource services to private sector. Another theme that was highlighted in hospitals included the profit-making aspect of the governmental sector in the form of drug supplying and profit-making retail.

**Conclusions:**

A change in health services after the enforcement of decentralisation was illustrated. Moreover, the incapacitation of public health systems and empowerment of the privatisation concept was the prevailing perception among stakeholders. Having contextualised in-depth studies and policy analysis in line with the global liberalisation and adjustment programmes is crucial for any health sector reform in Sudan.

## Background

“Decentralisation” in health is a concept that encompasses diverse anticipated objectives and associated consequences. WHO has categorised “decentralisation” into two types; functional and geographical decentralisation [1]. Likewise, this concept can be understood as transferring fiscal, administrative, ownership, or political responsibilities from the central ministry of health to local institutions in response to the health needs of local communities [2].The degree of decentralisation’ enforcement by authorities, usually aligns with the type of decentralisation, whether it be de-concentration, devolution, delegation, or privatisation. Furthermore, this is linked to the legal framework of the implementation within the country [1].

Sudan has been through several reforms of healthcare decentralisation from 1951 up until 1971. The aim of these reforms is to strengthen compatibility of the state government through delegation of authorities to medium and local administrative health units [3]. Moreover, in 1981 decentralisation was enforced as part of the broader economic and political liberalisation reforms of the structural adjustment programmes (SAP) [4]. The aim was to reduce the public expenditure on health, thereby, attempting to curb the economic recession [4]. The delegation of health authority from federal to local levels was further enforced with the intent to increase efficiency and access to health-service delivery [4, 5].

The health system in Sudan operates through three tiers: 1) The Federal Ministry of Health which is responsible for national health planning; 2) State ministries which are responsible for budget allocation and planning at the state level; and 3) the local health system for service delivery at the peripheries [3, 5]. Before 2011, health care delivery in Sudan comprised of tertiary and secondary level hospitals, which were under the authorization of Federal Ministry of Health (FMOH), as well as, district first referral level hospitals, which were under the authorization of state ministries. Primary health care was delivered through primary health care units, dispensaries, and health centers which are considered as the first referral units [5]. In contrast, after 2011, a radical reform of the health system was implemented and involved a subsequent shift of secondary and tertiary health care facilities to being under the complete control and authority of the state [6]. Services were transferred from large federal tertiary level hospitals, like Khartoum Teaching Hospital (KTH) and Jafaar Ibnaouf Hospital (JIH) located in the centre of Khartoum, to smaller district secondary hospitals. Nonetheless this late reform was not based on thorough research and did not consider the possible consequences in terms of the stakeholders’ perceptions. Several research studies from Uganda, China, and Nicaragua have reported decreased budget allocation for health services following decentralisation [7–9]. Successive fragmentation of the public health sector and private sector empowerment, after decentralisation, was also showcased in the Philippines and Argentina respectively [10, 11]. As shown in Ghana and Tanzania, the cuts associated with decentralisation lead to a decreased quality of and access to services. Likewise, the emplaced cost feed, drastically reduced the utilisation rate of public health services [9, 12].

This study explores perception of policymakers, community members, and health providers towards decentralisation implementation and its consequences on health services within Khartoum Locality, Sudan. This study can provide evidence needed, to redirect the consequences of the implemented policy toward positive outcomes, with better understanding to the local political market, especially at this time when Sudan has equality and human rights high on its political agenda.

## Methods

### Study design

**Fig. 1:**
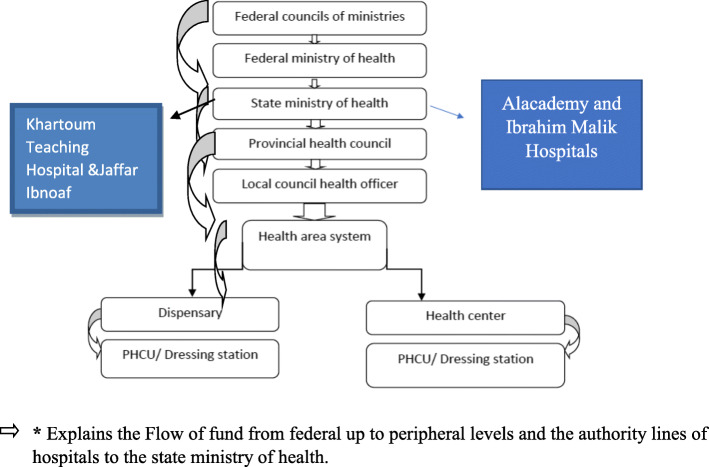
Health system structure and flow of funds. Explains the structure of of tri-layer health system as well as the Flow of fund from federal up to peripheral levels and the authority lines of hospitals to the state ministry of health

This study employed an exploratory qualitative method comprised of in-depth interviews and qualitative content analysis with an inductive approach [13]. The study was nested within a community-based survey, which involved a quantitative component. The aim is to understand the personal experiences and perceptions of stakeholders towards the decentralisation process and its effect on public health services (Fig. 1).

The study was conducted between July and December 2015 involving community members residing in the Khartoum Locality catchment areas. These areas are where decentralisation was implemented, as well as, have the presences of policy makers and health workers affiliated to decentralised hospitals.

### General setting

Sudan is a low middle-income country with a population of around 37 million, and 66.8% of the population resides in rural areas. Sudan has suffered from internal armed conflicts since its independence in 1956. The country is divided into 18 states and Khartoum is the main capital with the highest wealth among all of the states. However, poverty is widespread and the country’s foreign debt has reached $50 billion (61% of GDP) at the end of 2015 [14].

The distribution of health facilities shows disparities between the 18 states. Importantly the Khartoum and Gezira states alone, have 27% of the country’s public hospitals, 30.5% of private facilities, and 25% of PHC facilities [3, 6]. The adoption of the (SAP) in 1978 led to a prolonged reduction in health expenditure and the health budget deteriorated from 1.4% of GDP in 1987 to 0.24% in 1993 [4].

### Specific setting

The study was conducted in the Khartoum Locality (KL), which is one of Khartoum State’s seven localities, and it is composed of urban-suburban and rural populations, as well as, Internal Displaced Persons (IDPs). It contains 31 public health centres, 10 NGOs-led health centres, and 60 private health centres. Moreover, KL contains 13 hospitals, with three of them being multi-speciality general hospitals and ten being single speciality hospitals. There are also around 98 private hospitals, and 601 private clinics. KL has suffered the greatest extent of decentralisation among the seven localities. In 2012, health workers and service delivery were transferred from central hospitals such as Khartoum Teaching Hospital (KTH) and Jafar Ibnoaf Paediatrics Hospital (JIH), to two district hospitals called Ibrahim Malik Hospital (IBMH) and Alakademy Hospital (AKH).

### Selection and description of participants

**Fig. 2:**
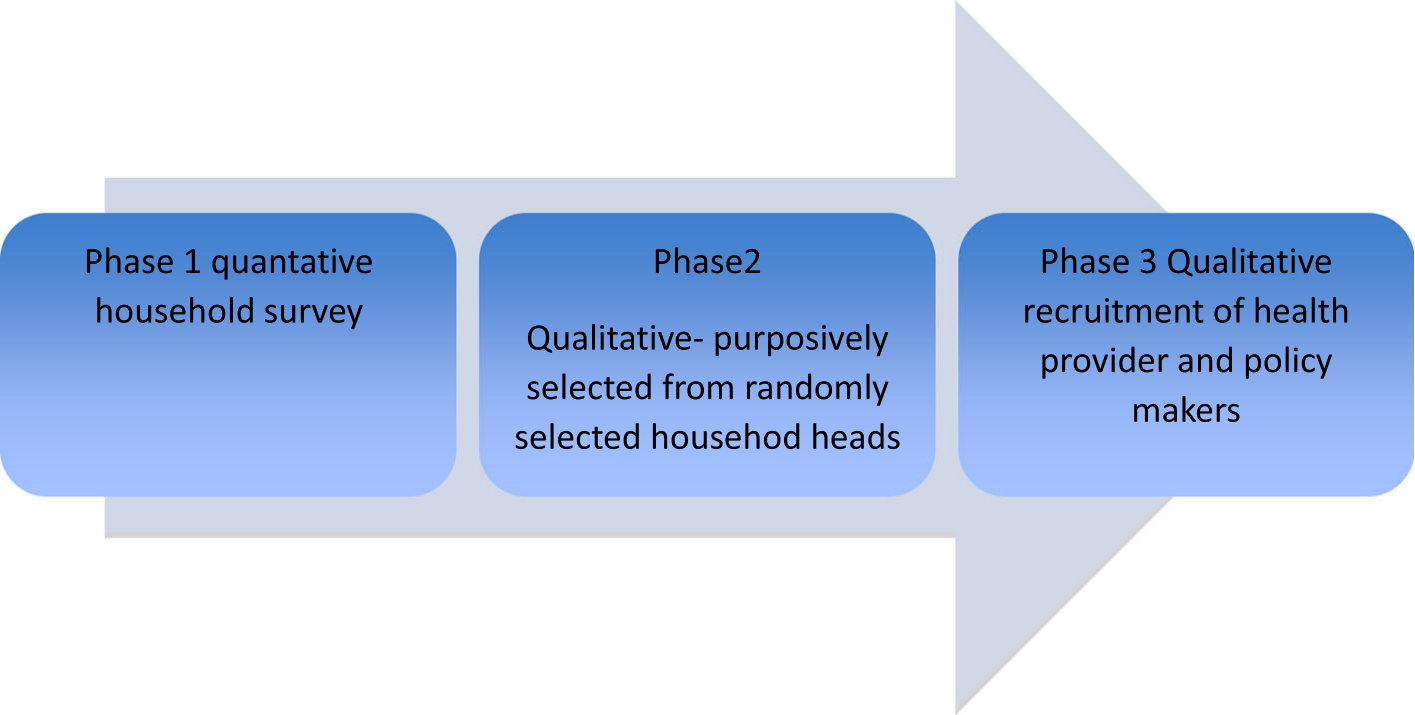
Syudy phases and timeliness. Illustrates the diiferent stages of the process of conducting the study from doing the household survey, to indepth interview with household heads to qualitative study with policy maker and health care providers.

This qualitative study had a stringent criterion for inclusion and exclusion of subjects. A total of 98 participants, all aged above 18 and including both males and females, were included. The selection criteria were aligned with the study aim of exploring the perception of stakeholders (community members in catchment areas of decentralised hospitals in KL, health care providers (HCP) in affiliated decentralised general and district hospitals of KL, and policymakers) on the implications of decentralisation. A sampling frame was determined and a matrix frame, including people residing in randomly selected households in the catchment areas of KL, was developed based on phase 1 quantitative data (Fig. 2). A total of 31 community members (CM) that experienced the perception of change in the delivery of health care after the process of decentralisation were recruited.

Two policymakers affiliated with FMOH and Khartoum State Ministry of Health (KMOH) were included. These policymakers are affiliated with the sector that implements decentralisation in the healthcare system (Table 1).

**Table 1:** Characteristics of study participants in Khartoum locality Sudan 2015

Characteristics	Number
**Sex**	
Male	43
Female	55
**Profession**	
Community member	31
Nurse	6
Lab technicians	7
Doctors	30
Pharmacists	9
Administrative staff	9
Statisticians	4
Policymaker at Federal Ministry of Health	1
Policymaker at Khartoum State Ministry of Health	1
**Facility Level**	
**Tertiary Hospitals**	
Khartoum Teaching Hospital	15
Jafar Ibnoaf Paediatrics Hospital	12
**Secondary-district**	
Ibrahim Malik Hospital	20
Alakademy Hospital	18

The health care providers (HCP) were recruited from four different central/tertiary hospitals (Khartoum Teaching Hospital, (KTH) Jafar Ibnoaf Paediatrics Hospital (JIP) and the district/secondary hospitals called Ibrahim Malik Hospital (IBMH), and Alakademy Hospital (AKH), which were decentralised in 2012. Nurses, lab technologists, doctors, and pharmacists were engaged. Men and women, as well as, different age groups were included (Table 1). HCP were selected randomly from hospital staff lists by taking into consideration the different professional and administrative categories. These included 30 medical doctors, 6 nurses, lab technicians, 9 pharmacists, 4 statisticians, and nine administrative staff members from tertiary and secondary facilities.

The study participants received written and oral information about the study and its objectives, as well as, any associated ethical issues. Participants were asked to sign a consent before the initiation of the study. Any non-consenting members were excluded from the study (Table 1). Saturation was determined when no more new themes or knowledge were generated from in-depth interviews, and it was reached at 98 participants.

### Data collection and entry

Data was collected through an in-depth interview using semi-structured topic-guides after obtaining consent from participants. The purpose of the interview guide was to obtain a variety of in-depth perceptions and experiences related to decentralisation. Interviewers were trained on communication skills, study protocol, conduction of in-depth interview. Tools were re-translated from English to Arabic, pre-tested and piloted as part of the practical training. Prolonged engagement was enforced to ensure familiarity of data collectors with the fieldwork and to build trust with study participants. Evaluation of interview process and peer review, as techniques were used to check validity and reliability of audios before transcription on day by day basis and to optimise process hence improve study findings credibility.

Participants were encouraged to reveal their thoughts, perceptions, and experiences on the implemented decentralisation reform in healthcare facilities.

Major themes of the interview guide included (see Arabic and English versions of interview notes):

- Study participants’ experiences about the process of decentralization implementation, decision-making, and availability of resources.
- Perception towards the decision and policy contents regarding decentralisation, as well as, the policies pertaining to the empowerment of the private sector.
- Perception of the implemented decentralisation and its effect on health provision.
- The participants’ experiences health services after the implementation of decentralisation.

Interviews were conducted in Arabic and lasted between 45 and 60 min. Transcripts were re-translated into English after transcription.

#### Data analysis

The analysis was aligned to inductive thematic content analysis. Local Arabic Sudanese accent was used and all of the interviews were audio-recorded. Transcription was the first stage of analysis and the research team conducted it to ensure the validity of the dialect. A general contextual overview was obtained through a thorough reading of the transcripts. Coding, categorisation, and generation of themes were generated subsequently. The organization of the interview data, thematic identification and analysis were carried out manually by two individuals and crosschecked. The anonymity of the participants was maintained and no identifiers were used to link any of the results to certain subjects. The researcher’s knowledge about the context enabled immersion strengthened the rigour in data collection and analysis.

#### Ethical consideration and informed consent

Ethical clearance was obtained from the National Research Ethics Review Committee (FMOH) in 2015 (FMOH ethical approval) and from REK (Regional Committee for Medical-Health Research Ethics) reference (2015/937/−REK), as well as, from Norwegian Social Science Data Services (NSD-44106/3/LB) (see NSD and REK ethical clearance). Participant information sheets were provided verbally and in a written manner. It clarified the study scope, objectives, major themes of the questions, anticipated duration of the interview, benefits of the study, and ethical aspects. Voluntary participation and possibility of declining to answer, as well as, the right to withdraw were highlighted to the participants. Signed informed consents were obtained before the interviewing.

Names and any traceable information were not physically recorded in the interviews. Moreover, only the researcher had access to the study data which was stored in a specific computer with password protection and no internet access.

### Trustworthiness

Involvement of diverse participants for the interview process enabled data source triangulation. Iterative probing and questioning, as well as, peer debriefing were used as tools to probe and discuss contradictions in the findings. Peer debriefing took place after each day of interviewing.

### Reflexivity

Subjective self-awareness of researcher’s position and the influence of the professional background on the selection of research topics and knowledge generation process, was considered [15]. Discussion, disclose, peer debriefing, debate and feedback meetings were conducted with research team during the preparatory phase.

## Results

Our analysis revealed four themes that were reported by study participants.

### Budget changes after decentralisation

Decline in the health budget after decentralisation was one of the major themes identified by participants. This austerity in public health expenditure is pointed out as the main feature of this decentralisation. There is a consensus among state-level policymakers that the unmet demand of the various states is due to the cuts in directed federal financial allocations. The allocation is around 200,000 SDG (30,300 USD), which only covers staff wages and part of the hospital operational costs. Hence, other hospitals’ needs are to be covered through hospital income-generating activities or by reshaping the spending such as terminating the programme “Free medicines in support of poor and vulnerable”.

Most health care providers pointed out that facility budget cuts varied from 15 to 30% attributing to gaps in the Khartoum state ministry of health funds. Khartoum state MOH had requested the Federal Ministry of Finance (FMOF) to postpone the budget cuts. As one health worker coined it: “In the beginning, the state ministry of finance said that they could not pay 15% of the budget. That was in the first year, and the FMOF had to pay all required amounts” Interviewee (46–25/11/2015). This decline in public health expenditure and allocation has challenged decentralized hospitals. This was outlined explicitly by administrative staff of Khartoum teaching hospital.” Subsequently and in between 2012 and 2015, the operational budget of KTH decreased to 400,000 SDG/month and JIH by 420,000 SDG/month” (Interview-67- HCP-21/12/2015).

A health care worker stated that the budget of IBMH increased from 80,000 to 120,000 SP/month after decentralisation and that the MOH budget for Revolving Drug Fund (RDF) free treatment of children under five was stopped in 2015. The allocated budget for emergency treatment was scarce and only composed of about 21,000/month. Hence, this caused an inability to cover free care for children under the age of five. On another occasion in December 2015 the hospital borrowed 20,000 SDG to buy emergency drugs. This deficit in the budget to cover medicines was also reported by one of the respondents: “The budget doesn’t cover even 25% of the drug orders due to the huge number of patients” (Interview-57-16/11/2015).

Under this theme, certain healthcare staff stated that the main aim of decentralisation was to decrease the state support for public healthcare, in particular hospital services. Others mentioned that this deficit hindered the equipment’s maintenance and renovation, which subsequently led to hospital debts.

Hospital debt settlement used to be the responsibility of FMOH and this is now upheld after decentralisation. This is highlighted by the following statement of an interviewee: “Accumulated hospital debts were usually covered by FMOH; nonetheless, this was stopped after decentralization” (Interview-63-25/12/2015, 46–25/11/2015).

Shortfalls in the budget were denoted as a serious consequence of decentralisation implementation. Thus, this negatively impacted the decentralised hospitals and reduced the budget for free treatment. Therefore, this led to numerous privatisation policies which placed a massive burden on patients.

#### Privatisation of the medicine supply system

The heavy emphasis on privatisation of medicine supply was one of the main perceived consequences of decentralisation among the interviewed health care providers (HCP). Some HCPs explained that after the decentralisation the Revolving Drug Fund (RDF) (Khartoum state drug supply agency) started adding an extra 5% to the prices of drugs and supplies, which increased profit margins. The ministerial decree in 1991 [16] stated that the appointed National Medicine Supply fund (NMSF) should be the main supplier for all public hospitals. However some decentralised facilities recognised that they were only able to receive their supply needs from RDF and not through a direct channel from NMSF.

“Drugs are supplied through RDF although they are cheaper if directly purchased from NMSF, yet we are forced to get drugs from RDF” (Interview-57-6/11/2015).

“I perceive that some political agenda is behind this decentralisation that aims to weaken the role of NMSF as the only supplier for medicines and equipment” (Interview-51-25/11/2015).

“RDF gets supplies from private pharmaceutical companies despite the availability of the same item at NMSF and of course at a cheaper price” (Interview-51-25/11/2015).

Furthermore, the interviews also highlighted a novel perception linked to the privatisation of medicine financing due to decentralisation. An HCP stated that the “NMSF” was transferred to an autonomous institution and was no longer under the authority of the government’s “Audit Office”. Thus, this meant that a step closer to privatisation. Moreover, certain HCP’s also highlighted that after decentralisation, the KMOH took charge of internal hospital pharmacies and converted them to “for-profit retail pharmacies” which sell medicines to the general public.

### Weakening of the public health sector

Many community members and health care providers stated that the aim of decentralisation was to debilitate Khartoum teaching hospital and Jafar Ibnoaf paediatrics hospital, thereby, causing patients to turn towards the private health sector.

*“*The aim of decentralization is to outsource the central-hospitals for the benefit of private sector. Worth telling that many of the regime affiliated people are investors in health and they own private hospitals”. (Interview-3-3/10/2015).

Numerous community members argued that patients from states other than Khartoum are also forced to turn towards the private sector because of the low capacity present at peripheral facilities. Unavailability of drugs, inadequate services, and delays in treatment are all reasons why citizens prefer private sector. Moreover, the issue of selling the land of the Khartoum teaching hospital and its central posture to investors has appeared as a factor that benefited the private sector, as stated by community members and health care providers (Interview-22-18/10/2015). Many community members and health care providers argued that the process of decentralisation implementation, as well as, the sudden closure of emergency services without community involvement led to patients preferring the private sector. Furthermore, healthcare staff members left the public facilities to work in the private sector.

Some flows in the practice appeared after the decentralisation of emergency services out of Jafar Ibnoaf (JIH). Some patients sought care with private specialists affiliated with JIH; hence, they were directly referred to the hospital and bypassed the peripheral facilities.

Some community members stated that the direct consequence of decentralisation was to indirectly empower the private sector and force people to access these hospitals for all cases, including emergencies. In addition, the transfer of services led to the fragmentation of public services. In contrast, the private sector evolved into a comprehensive service provision. Other reasons that forced citizens to access the private sector included the public sector not containing resources such as an intensive care unit and newborn facilities.

“Since the closure of Jafar Ibnoaf Paediatrics hospital, we decided not to go to any public hospital. We only go to private clinics”. (Interview-12. 9/10/2015).

#### Privatization of public health care services

Many HCPs stated that after decentralisation the hospitals started to decrease the number of patients in the list of morning surgical operations and increased evening list, which was far more expensive. Moreover, cost recovery schemes were introduced such as the Laboratory service provision in AKH, which was carried through as a partnership between the MOH and a private entity. Likewise, the same applied for other services since the study participants stated that before decentralisation, the emergency surgical operations used to be free at AKH and IBMH. However, after decentralisation the patients were getting charged. Budget cuts associated with decentralisation in JIH has affected some aspects such as children’s investigations and medicines becoming commoditised and free meals for children being scrapped. (Interview-12 10/10/2015). Some CM and HCPs mentioned that “even basic aids and materials (Cotton, gauze, plaster, drugs, soap and sutures) are to be bought out of pocket by the patients.” (Interview-8. 7/10/2015).

Health care providers stated that delivery fees increased to 270,000 after decentralisation. Furthermore, ergometrine, which is an emergency medicine used in normal labour had to be purchased by patients themselves (Interview-62–7/12/2015).

“Fee exemption policy for low-income groups is withheld after decentralisation which was mainly due to associated budgets cuts” (Interview 46–25/11/2015). One of the HCP mentioned that in AKH, staff members received a memorandum to not cover those groups. The profit generated from medicine sales at internal pharmacies was used to cover service provision for low-income people or those with unknown ID. This has stopped after decentralisation led to the transfer of the pharmacies authority to RDF.

## Discussion

This study is conducted as a critique for the health system in Sudan mainly to elucidate perceived effect of decentralisation implementation on health care services. The majority of participating community and healthcare workers considered implemented decentralisation as debilitation of public health services and invigoration of the private health sector. Comprehension of the overall political context of Sudan is fundamental in understanding perceived consequences of decentralisation [17].

The low allocation and the budget cuts is situated within the structural adjustment program’s remedy (SAP) that has been imposed to Sudan in 1978, as part of The World Bank (WB) and International Monetary Fund (IMF) treaty. All radical reforms and its correlated profit-making schemes have shaped the implementation of decentralization towards weakening public sector, facilitation for private sector, and commodification of health. This is highlighted in the case of Latin America, where the free market economy and privatization has slipped under the guise of the decentralization of health services. It has also lead to an increase in the number of poor groups who lost their “agency” to accessing public health services, as they were enforced to seek health in the private sector to receive a comprehensive package of services [8]. A study from Bolivia explored the affinity between health services privatisation and SAPs implementation [18].

Revenue generation might be associated to decentralisation and its transfer of power and authority to local levels [17].. Study participants have elucidated many forms of privatisation and profit making-schemes such as cost recovery, and public-private partnership being imposed as part of the routine functionalities of some lab services. Furthermore to that is the transformation of internal public hospital pharmacies to becoming for-profit retail-pharmacies. This also occurred in Nicaragua, where decentralisation has led to the creation of private wards within public hospitals [8]. Patients, as main source of health expenditure in Sudan, steadily cover such implications through out of pocket payments.

A recent study has shown a very high out of pocket expenditure that reached up to 95.94% in 2013 [19]. This puts individuals & households under catastrophic events and discards the essence of welfare and health as being a fundamental human right [19]. It also implies several challenges to meeting population needs and developments [20]. The commodification of health services consequently opens up new spaces for capital accumulation and extraction of the surplus-value [20].

This goes in hand with the contextual impacts of decentralisation implementation in Uganda, Ghana, and Tanzania, where far fewer people were able to use the health services [9, 12, 21].

The lack of sufficient investment in public health services could lead to the inadequacy of capacities such as lack of adequate infrastructure, unavailability of drugs, and delayed treatment. Hence, this, directly and indirectly, enabled the private sectors to flourish. The fragmentation of health services, shortages in ICU and new-born services after decentralisation implementation in the Philippines resulted in patients turning to the private sector [11].

Vulnerable populations such as internally displaced people and those living in the peripheries of the Khartoum locality might be forced to access private sector due to unavailability of emergency services after decentralisation.

A central theme highlighted by this study was the privatisation of maternal and child services, which was also revealed in a comparative study between Indonesia and Kenya [22].

This has led to communities having to pay for various stages of healthcare such as treatment, meals, and emergency medicines. Internal pharmacies stopped supporting free treatment of vulnerable-groups after being transferred to the RDF. These changes in the drug supply system have been supported by other literature from Sudan [23, 24].

The study findings show that there is a decrease in the hospital budget and complete termination of treatment for children and adults, thereby, facilitating further support for the private sector. This finding has been supported by several studies that favours privatisation of national medicine supply, which is also in line with the WB recommendations and economic liberalisation policy [25].

The continued effect of SAP on budget reduction emerged since its signature in 1980.The per capita health budget had decreased from 1.4SDG in 1986/87 to 0.24SDG in 1993/94 [4].. This has directly afflicted the implementation of user fees and the cost-sharing mechanisms as alternative health financing.

Federal decentralisation was implemented to overcome the scarcity of resources by transferring responsibilities to the state and peripheries [3]. Hence, the main spending will be sorted by people spending out of their own pocket to finance health services within the context of SAP. This clearly augments the privatisation policy.

The implementation of neoliberal policies and market schemes has taken place across the globe, thereby, causing an increase in population inequality in terms of accessing health services. Moreover, it has also led to the health system deteriorating, working conditions becoming poorer, and health outcomes declining. The budget cuts have included many social services such as education, water, electricity, and environmental sanitation. In addition, removal of subsidises and wage cuts, as well as, the devaluation of local currency and decrease in public sector employees has led to a pronounced decline. These policies have been implemented through the World Bank and IMF by direct negotiation with the countries and through the utilisation of rescheduling debts and providing loans [26].

All, these policies led to a decrease in income which affected the vulnerable population groups. Furthermore, the expansion of market schemes was supported by the withdrawal of the state from delivering social services [27]. The role of NGOs was designed to mitigate the catastrophic effect of these policies on the poor. This NGO model of health service delivery was described as covering privatisation in Mongolia [28]. Another study from Costa Rica argued that the concept of decentralisation, private/public partnership, community participation, and civic society has invaded the discussion regarding the development of transferring the cost of health services to communities [29].

The main strengths of our study include the involvement of 98 participants from different categories. Hence, this increased the credibility of our findings through triangulation of data sources [30].

The limitations of the study were related to sensitivity and the political dimension of the topic caused certain reluctance. Also, the subjectivity nature of the qualitative methodology led to involving the process of knowledge generation, which affected the validity of the findings.

## Conclusion

This study revealed perceptions of change after implementation of decentralisation in term of weakening of public sector. The study highlighted strong indications of healthcare budget cuts and privatisation of drug supply systems after the implementation of decentralisation. Moreover, this led to internal hospital pharmacies focusing on profit-making and on removal of free treatment schemes for vulnerable populations. The budget cuts and deficits resulted in deterioration of quality and capacity of decentralised hospitals, thereby, facilitated privatisation.

The process of implementation took place in a setting where structural adjustment program packages have already embraced privatisation. Understanding influential contextual factors is crucial in the process of decentralization implementation to achieve direct policy consequences.

In-depth policy analysis and evidences are needed to lead any health sector reform in Sudan.

## Data Availability

All relevant data is included in the manuscript, but it cannot be available publicly in a separate repository to avoid compromising confidentiality or any ethical standards due to political sensitivity of data.

## Abbreviations

KTH: Khartoum teaching hospital
JIH: Jafaar Ibnaouf hospital
IBMH: Ibrahim Malik hospital
AKH: Alakademy hospital
WHO: World health organization
SAP: Structural adjustment program
FMOH: Federal ministry of health
GDP: Gross domestic product
PHC: Primary health centre
KL: Khartoum locality
IDPs: Internally displaced person
PHCU: Primary health care unit
KMOH: Khartoum State ministry of health
CM: Community members
HCP: Health care providers
REK: Regional committee for medical-health research ethics
NSD: Norwegian social science data services
SDG: Sudanese pound
USD: United States dollar
FMOF: Federal ministry of finance
RDF: Revolving drug fund
NMSF: National medicine supply fund
WB: World bank
IMF: International monetary fund
ICU: Intensive care unit
NGO: Non- governmental organization
